# Tropomyosin receptor kinase (TRK) biology and the role of *NTRK* gene fusions in cancer

**DOI:** 10.1093/annonc/mdz383

**Published:** 2019-11-18

**Authors:** A Amatu, A Sartore-Bianchi, K Bencardino, E G Pizzutilo, F Tosi, S Siena

**Affiliations:** 1 Department of Hematology and Oncology, Niguarda Cancer Center, Grande Ospedale Metropolitano Niguarda, Milan; 2 Department of Oncology and Hemato- Oncology, Università degli Studi di Milano, Milan, Italy

**Keywords:** TRK, tropomyosin receptor kinase, *NTRK* gene fusions, TRK fusion cancer

## Abstract

The tropomyosin receptor kinase (TRK) family of receptor tyrosine kinases are encoded by *NTRK* genes and have a role in the development and normal functioning of the nervous system. Since the discovery of an oncogenic *NTRK* gene fusion in colorectal cancer in 1986, over 80 different fusion partner genes have been identified in a wide array of adult and paediatric tumours, providing actionable targets for targeted therapy. This review describes the normal function and physiology of TRK receptors and the biology behind *NTRK* gene fusions and how they act as oncogenic drivers in cancer. Finally, an overview of the incidence and prevalence of *NTRK* gene fusions in various types of cancers is discussed.

**Key Message**

TRK fusion proteins, which are encoded by *NTRK* gene fusions, are oncogenic drivers in many cancers. An understanding of the oncogenic mechanism behind the TRK fusion proteins expressed by these gene fusions and the prevalence of TRK fusion-positive cancers is critical to providing optimal targeted therapy.

## Introduction

The identification of gene fusions in a variety of cancers has provided actionable targets that have expanded therapeutic options and facilitated precision medicine. These gene aberrations result in the expression of fusion proteins with constitutive activity that become oncogenic drivers [1]. The tropomyosin receptor kinase (TRK) family of receptor tyrosine kinases are of interest as the *NTRK* genes that encode them are involved in gene fusions identified in a wide range of adult and paediatric tumours. 

In this review, we discuss the normal function and physiology of TRK receptors, the biology behind *NTRK* gene fusions, the mechanisms by which *NTRK* gene fusions become oncogenic drivers in cancer, and the incidence and prevalence of *NTRK* gene fusions in a variety of cancers.

### Normal function and physiology of *NTRK* genes and TRK receptors

#### Structure

**Figure 1.**
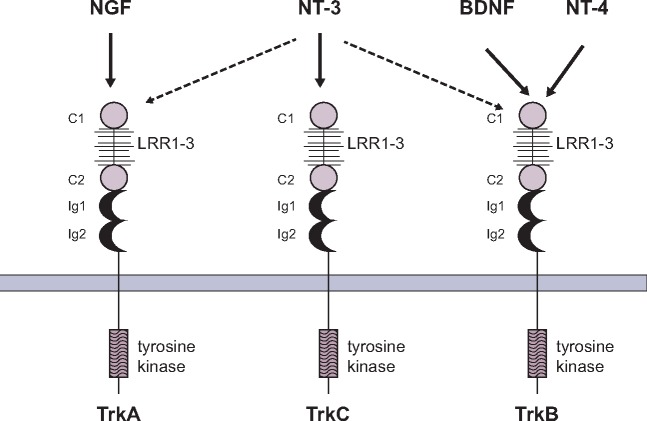
Structure of TRK receptors and interaction with ligands [5]. The neurotrophins display specific interactions with the three TRK receptors: NGF binds TRKA, BDNF and NT-4 bind TRKB and NT-3 binds TRKC. NT-3 can also activate TRKA and TRKB albeit with less efficiency. BDNF, brain-derived neurotrophic factor; C1/C2, cysteine-rich clusters; Ig1/Ig2, immunoglobulin-like domains; LRR1–3, leucine-rich repeats; NGF, nerve growth factor; NT-3/4, neurotrophin 3/4; TRK, tropomyosin receptor kinase.

TRKA, TRKB and TRKC are transmembrane proteins that comprise the TRK receptor family. TRKA is encoded by the *NTRK1* gene located on chromosome 1q21-q22 [2]. TRKB is encoded by the *NTRK2* gene located on chromosome 9q22.1 [3]. TRKC is encoded by the *NTRK3* gene located on chromosome 15q25 [4]. Each of the TRK receptors consists of an extracellular domain, a transmembrane region and an intracellular region containing the tyrosine kinase domain. The extracellular domain contains a cysteine-rich cluster (C1) followed by three leucine-rich 24-residue repeats (LRR1–3), another cysteine-rich cluster (C2) and two immunoglobulin-like domains (Ig1 and Ig2; Figure 1) [5–7]. The LRR1–3 motifs are specific to TRK proteins and are not found in other receptor tyrosine kinases [6]. The intracellular region contains five key tyrosine residues (Figure 1): three within the activation loop of the kinase domain, which are necessary for full kinase activity, and two on either side of the tyrosine kinase domain, which serve as phosphorylation-dependent docking sites for cytoplasmic adaptors and enzymes [8].

#### TRK receptors and associated ligands

**Figure 2.**
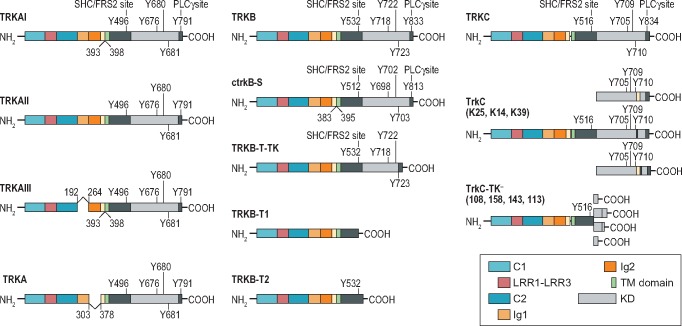
Known splice variants of TRKA, TRKB and TRKC [6]. C1/C2, cysteine-rich clusters; Ig1/Ig2, immunoglobulin-like domains; KD, kinase domain; LRR1–3, leucine-rich repeats; TM, transmembrane; TRK, tropomyosin receptor kinase.

The TRK receptors are activated by a family of four proteins called neurotrophins. Neurotrophins were initially identified as survival molecules for sensory and sympathetic neurons [9], but are now understood to play many roles in the development and function of the nervous system [10]. Each of the four neurotrophins have specificity for a particular TRK and bind to it with high affinity (Figure 1). Nerve growth factor (NGF) binds to TRKA [11, 12], both brain-derived neurotrophic factor (BDNF) and neurotrophin 4 (NT-4) bind to TRKB [13–15] and neurotrophin 3 (NT-3) binds to TRKC [16]. NT-3 can bind to all three TRK receptors but has highest affinity for TRKC and is its sole ligand [14, 15, 17, 18]. Alternative splicing of TRK proteins can alter the interaction between a TRK receptor and its specific neurotrophin (Figure 2) [10, 19]. For example, short amino acid sequence insertions observed in the juxtamembrane region of the extracellular domains of TRKA and TRKB enhance their binding with non-cognate ligands [20, 21]. Isoforms of TRKA and TRKB that lack this insertion are activated strongly only by NGF and BDNF, respectively. In contrast, with this insertion, the TRKA splice variant is activated by NT-3 in addition to NGF [20] and the TRKB splice variant is readily activated by NT-3 and NT-4 in addition to BDNF [21]. Alternative splicing of exons encoding parts of the intracellular domains of TRK receptors may also affect downstream signalling initiated by neurotrophin binding to the receptor. Such alternatively spliced TRKB and TRKC isoforms have been observed to contain comparatively short cytoplasmic motifs missing the tyrosine kinase domain, leading to a lack of receptor response to neurotrophins [22]. For example, alternative splicing of the *NTRK3* gene may lead to amino acid insertion into the TRKC tyrosine kinase domain, which in turn results in modified kinase substrate specificity and impaired ability to promote neuronal cell differentiation [23].

#### Normal TRK signalling pathway

**Figure 3.**
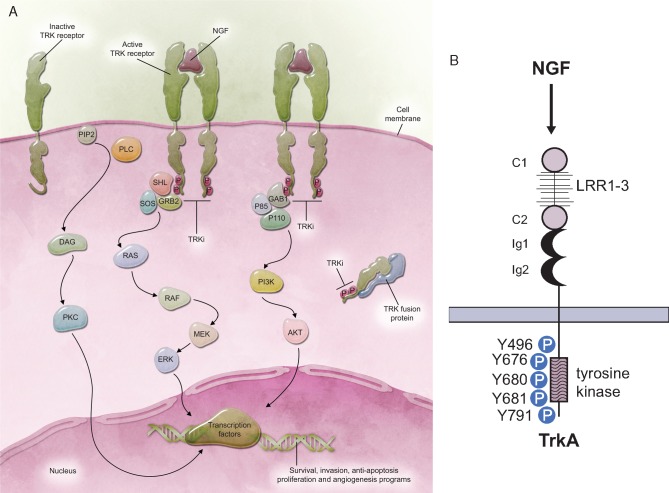
TRK signalling pathway. (A) Overview of TRK signalling pathway; (B) activation of TRKA. C1/C2, cysteine-rich clusters; Ig1/Ig2, immunoglobulin-like domains; LRR1–3, leucine-rich repeats; NGF, nerve growth factor; TRK, tropomyosin receptor kinase, TRKi, tropomyosin receptor kinase inhibitor.

The TRK signalling pathway is initiated when neurotrophin binding to TRK receptors at the cell surface causes the formation of receptor dimers (Figure 3A). The dimerised receptor autophosphorylates specific tyrosine residues in the activation loop of the kinase domain [Y676, Y680 and Y681 in TRKA (Figure 3B) and the corresponding residues in TRKB and TRKC] [8]. This phosphorylation is required for activation of the TRK receptor [6] and leads to subsequent phosphorylation of additional tyrosine residues (Y496 and Y791 in TRKA), enabling docking of cytoplasmic adaptors and enzymes [5–7], which in turn drives a variety of downstream signalling pathways [6]. The binding of TRKA by NGF causes activation of the RAS/MAPK pathway, leading to increased cellular proliferation and growth via ERK signalling [24, 25]. Neurotrophic binding to TRKB results in activation of the RAS-ERK, PI3K and PLCγ pathway, resulting in neuronal differentiation and survival [24, 25]. TRKC binding to NT-3 causes preferential activation of the PI3K/AKT pathway, preventing apoptosis and increasing cell survival [24, 25].

#### Role in development and physiology

TRK receptors are predominantly expressed in neuronal tissue and play an essential role during embryonic development as well as in the normal function of the nervous system [7, 26]. The activation of TRK receptors by neurotrophins has an impact on a variety of neuronal events, such as neuronal cell differentiation and survival, cell proliferation, synaptic formation and plasticity, membrane trafficking, and axon and dendrite formation [7, 19, 27].

TRK receptors and their respective neurotrophins have been implicated in the survival and differentiation of sensory ganglia. TRKA receptors are expressed in almost all nociceptive neurons in the dorsal root and trigeminal ganglia [28, 29], while dorsal root ganglia neurons that differentiate in proprioceptive neurons start expressing TRKC during neurogenesis. Neurons in the nodose-petrosal ganglion, which conveys visceral sensory information about blood pH and pressure, express TRKB and are dependent on BDNF for development and differentiation [30, 31].

TRK receptors and their respective neurotrophins have been implicated in memory formation and retention, nociception and proprioception [31, 32], as well as having roles in non-neuronal tissues including the vasculature, ovaries and immune system [33–36]. Loss-of-function mutations in *NTRK* genes can result in several diseases, indicating the role of TRK receptors in normal regulation and function. TRKA receptors are involved in pain sensation; loss-of-function mutations in TRKA are observed in class IV hereditary sensory and autonomic neuronal disorders (such as congenital insensitivity to pain with anhidrosis), which result in impaired ability to sense differences in temperature or feel pain [37, 38]. Loss-of-function mutations in TRKB result in energy imbalances, loss of appetite control and subsequent obesity, in addition to defects in learning, memory and nociception [39–41].

### Discovery of aberrant gene fusions and ligand-independent oncogenic proteins

#### Discovery of *NTRK* gene fusions in cancer

**Figure 4.**
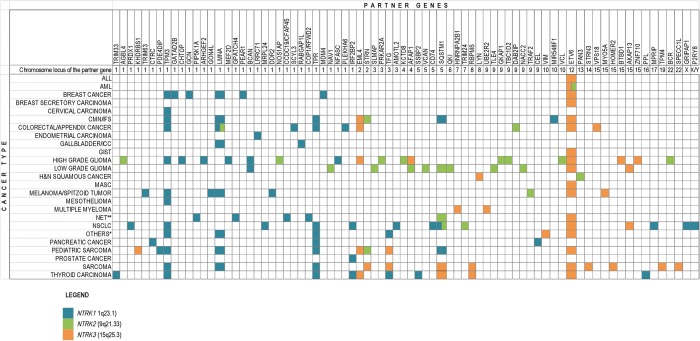
*NTRK* gene fusions in cancers. *Sinonasal low-grade non-intestinal-type adenocarcinoma, parotid gland acinic cell carcinoma, anaplastic thyroid carcinoma, Erdheim–Chester disease, interdigitating dendritic cell sarcoma. **One large-cell neuroendocrine carcinoma of the lung with *COP1-NTRK1*, one small-cell lung cancer with *ETV6*-*NTRK3.* CMN, congenital mesoblastic nephroma; GIST, gastrointestinal stromal tumour; ICC, intrahepatic cholangiocarcinoma; IFS, infantile fibrosarcoma; MASC, mammary analogue secretory carcinoma; NET, neuroendocrine tumour; NSCLC, non-small-cell lung cancer.

Somatic fusions involving the *NTRK* genes were first observed in a patient with colorectal cancer (CRC) in 1986, when Martin-Zanca et al. identified a chimeric fusion oncogene resulting from an intrachromosomal rearrangement at 1q22-23 [42]. This oncogene involved the tropomyosin 3 gene (*TPM3*) and a locus that was subsequently cloned and found to encode a high-affinity NGF receptor (*NTRK1*) [12]. Following the discovery of this *TPM3-NTRK1* gene fusion, the identification of other *NTRK* gene fusions in CRC [43–45] triggered the interest of clinicians in the possible existence of oncogenic gene fusions in other types of cancers; to date, over 80 different fusion partner genes have been identified in a wide array of tumours (Figure 4).

#### Oncogenic mechanism of *NTRK* gene fusions

In *NTRK* gene fusion events, the 3′ region of the *NTRK* gene is joined with the 5′ end of a fusion partner gene, either by intrachromosomal or interchromosomal rearrangement. The resulting fusion gene encodes a protein containing the N-terminus of the fusion partner joined to the C-terminus of the TRK protein, including the catalytic tyrosine kinase domain [27]. The majority of characterised *NTRK* gene fusions contain a 5′ partner gene sequence encoding one or more dimerisation domains. These domains mediate the corresponding constitutive tyrosine kinase activity that occurs, thus conferring ligand-independent oncogenic potential through uninterrupted downstream signalling messages, promoting cell proliferation and survival [27].

### Incidence and prevalence of *NTRK* gene fusions

Incidence and prevalence data for *NTRK* gene fusions have only recently become clearer following the increasing availability of next-generation sequencing (NGS) and comprehensive molecular testing methods. *NTRK* gene fusions have been identified in two main categories of tumours with vastly differing rates of occurrence; certain rare cancers present with a high frequency (>80%) of *NTRK* gene fusions, while some more common cancers present with a lower frequency of *NTRK* gene fusions (<25%) [24, 27, 46]. *NTRK* gene fusions have been estimated to occur in up to 1% of all solid tumours [27, 46, 47]. Gene fusion events appear to arise more commonly in the *NTRK1* and *NTRK3* genes, with the possible exception of brain tumours [27, 46–48]. Immunohistochemistry (IHC) screening in 1043 various solid tumours showed TRKA expression in 1.6% of samples, including CRC, lung cancer, biliary tract carcinoma and thyroid cancer. Of note, only 5.9% of these showed *NTRK* gene rearrangements, while 88.2% of cases displayed *NTRK1* gene copy number gain without amplification [49]. In a retrospective analysis of 33 997 patients, screening with a targeted DNA-based NGS panel (MSK-IMPACT) identified 87 patients (0.26%) with oncogenic *NTRK1–3* gene fusions. The prevalence of *NTRK1–3* gene fusions in this group ranged from 0.13% to 17.7% depending on the various tumour types. Screening with pan-TRK IHC in this study showed better sensitivity than DNA-based NGS (87.9% versus 81.1%) but reduced specificity (81.1% versus 99.9%) [50].

#### *NTRK* gene fusions are pathognomonic in certain rare paediatric and adult cancers

Infantile fibrosarcoma (IFS), a malignant tumour of fibroblasts, represents <1% of all paediatric cancers but is the most commonly occurring non-rhabdomyosarcoma soft tissue sarcoma in children under 1 year of age [47]. IFS is virtually identical histologically to the cellular variant of congenital mesoblastic nephroma (CMN), an infantile spindle cell tumour of the kidney that occurs in the same age group and represents ∼5% of all childhood renal neoplasms. In 1998, Knezevich et al. discovered a recurrent *ETV6*-*NTRK3* gene fusion in IFS, which was found to occur in ∼70% of cases of IFS [51]. The same year, two other groups identified the same *ETV6*-*NTRK3* gene fusion in the cellular variant of CMN, establishing a genetic link between IFS and cellular CMN [52, 53]. Thereafter, identification of the *ETV6*-*NTRK3* translocation has become a useful diagnostic marker for IFS/CMN, and the presence of this gene fusion is considered pathognomonic for these two rare cancers. Several additional novel translocations involving *NTRK* genes have subsequently been described in IFS/CMN [54, 55] (Figure 4); consequently, genomic testing using break-apart fluorescence *in situ* hybridisation specific for *ETV6* may be insufficient both as a diagnostic and predictive marker [56].

Secretory breast carcinoma (SBC) is one of the rarest types of breast carcinomas, accounting for ∼0.15% of all breast cancers [57]. It is characterised by intracellular and extracellular eosinophilic secretions and usually presents as a triple-negative breast carcinoma with an immunohistochemical profile akin to basal-like breast carcinoma. Tognon et al. first reported an *ETV6*-*NTRK3* gene fusion in 12 out of the 13 cases of SBC by identifying the corresponding chromosomal translocation t(12; 15)(p13; q25) [58].

Mammary analogue secretory carcinoma (MASC) is a rare neoplasm of minor and major salivary glands morphologically and immunohistochemically similar to SBC. Since it was first described in 2010 by Skálová et al. [59], fewer than 300 cases have been reported in the literature [60]. Skálová et al. found that of 14 cases of MASC, all but one was characterised by the *ETV6*-*NTRK3* gene fusion [59]. While *ETV6*-*NTRK3* is the most common gene fusion seen in MASC, other rearrangements involving *ETV6* and *NTRK1* or *NTRK2* have been identified [53]. On the other hand, no partner genes other than *ETV6* have been described in cases of MASC harbouring *NTRK3* rearrangement (Figure 4).

#### *NTRK* gene fusions in common cancers

##### Thyroid cancer

Papillary thyroid carcinoma (PTC) is the most common type of thyroid cancer, accounting for 80% of all thyroid cancer cases [61]. Since the identification of *NTRK1* as an oncogenic driver in PTC by Bongarzone et al. in 1989 [62], the reported frequency of *NTRK1* rearrangement in PTC has been shown to range from <5% to 25% [63–68]. More recently, novel *NTRK3* fusion genes have been discovered in PTC, with *ETV6*-*NTRK3* being the most common rearrangement found after any *RET-PTC* isoform in The Cancer Genome Atlas Project [61]. While the prevalence of *ETV6*-*NTRK3* in PTC in adults is very low (1%), it is the second most common rearrangement seen in radiation-associated PTC [69, 70]. 

##### Colorectal and appendiceal cancer

Following the identification of *TPM3-NTRK1* as an oncogenic driver in CRC in 1986 [42], the third most common form of cancer, nothing further was reported about this gene fusion until almost 30 years later when Ardini et al. characterised the *TPM3-NTRK1* rearrangement at the genomic level for the first time, finding that the observed breakpoint within exon 8 of *NTRK1* in CRC differed from those previously identified for the *TPM3-NTRK1* gene fusion in PTC. This group also developed and validated an IHC method for the identification of TRKA-positive clinical specimens, offering a readily applicable approach to screening CRC for TRKA overexpression and thus identifying those cases that could potentially benefit from targeted therapy [43]. Further cases of CRC harbouring either *NTRK1* or *NTRK3* gene fusions involving different partner genes have subsequently been reported and, in some cases, demonstrated pharmacologically actionable (Figure 4) [44, 45, 71–73]. A recent molecular profiling study used a plasma-based cell-free circulating tumour DNA NGS assay to detect gene fusions in 4290 patients with CRC. Using different gene panels, including one testing for *NTRK1* (but not *NTRK2* or *NTRK3*) gene fusions, only three (0.07%) cases were detected [74]. These data are consistent with the prevalence previously found using a tissue-based NGS assay [75]. Notably, gene fusions seem to be associated with high mutation burden [74], and microsatellite instability (MSI) is frequently found in CRCs harbouring *NTRK* gene fusions [44, 71, 76]. Hypothetically, the increased mutational frequency in MSI-high CRCs could explain the higher incidence of *NTRK* gene rearrangements as well as *NTRK* mutations [77]. To date, only *NTRK2* fusions have been identified in cases of appendiceal adenocarcinoma [73, 78].

##### Lung cancer

Lung cancer is the leading cause of cancer-related mortality in the world. Non-small-cell lung cancer (NSCLC) is the most common type of lung cancer, accounting for 85% of all lung cancer cases [79]. *NTRK1* gene rearrangements in NSCLC were first described in 2013 among a subset of patients with NSCLC with adenocarcinoma histology and no detectable *EGFR*, *KRAS*, *ALK* or *ROS1* alterations (3/91; 3.3%) [80]. In a larger and unselected cohort of 1378 patients with NSCLC, *NTRK1* gene fusions were detected in two patients (0.1%) [81]. *NTRK2* and *NTRK3* gene fusions in NSCLC have also been described [48, 82]. Overall, *NTRK* gene fusions occur at a frequency of ∼0.1%−1.0% [27, 80, 81] (Figure 4).

##### Sarcoma

*NTRK* gene fusions are relatively rare in soft tissue sarcoma. Testing on 1272 soft tissue sarcoma samples identified eight cases (<1%) with *NTRK1* or *NTRK3* gene fusions, with one-half of these found in patients under the age of 5 years [83]. Recurrent *NTRK1* gene fusions have been noted in soft tissue sarcomas characterised by a prominent myopericytic/haemangiopericytic growth pattern [84]. Several studies involving the genetic sequencing of tumour samples have led to the characterisation of novel subtypes of sarcoma not previously described. Undifferentiated uterine sarcoma is a diagnosis of exclusion after more common uterine mesenchymal tumours, such as leiomyosarcoma, have been ruled out. From a database of gynaecological cancer patients, Chiang et al. prospectively identified four *NTRK* gene fusion-positive undifferentiated uterine sarcomas with spindle cell morphology that were morphologically and immunophenotypically unique from leiomyosarcoma and other undifferentiated uterine sarcoma. This discovery suggested a novel uterine sarcoma subtype defined by the presence of recurrent *NTRK* gene fusions [85]. Similarly, Agaram et al. described a novel and distinct subset of *NTRK1* gene fusion-positive soft tissue tumours occurring in children and young adults resembling lipofibromatosis (LPF) but displaying cytologic atypia and a neural immunophenotype. These tumours have been provisionally named LPF-like neural tumours and are defined by *NTRK1* oncogenic activation [86]. *ETV6*-*NTRK3* gene fusions have also been identified in inflammatory myofibroblastic tumours in adolescent and adult patients [87], especially in *ALK*-negative tumours [88, 89].

##### Central nervous system cancers

*NTRK* gene fusions have been identified in both paediatric and adult primary central nervous system (CNS) tumours, including glioblastoma multiforme (GBM), paediatric gliomas and astrocytomas [27]. Frattini et al. analysed 185 samples of GBM and discovered 2 *NTRK1* gene fusions (1%) with two different 5′ fusion partners (*NFASC-NTRK1* and *BCAN-NTRK1*) [90]. Several additional *NTRK* translocations have subsequently been described in GBM (Figure 4). In a series of 127 paediatric high-grade gliomas (HGGs), Wu et al. reported recurrent fusions involving *NTRK* genes in 4% of diffuse intrinsic pontine gliomas and 10% of non-brainstem HGGs (NBS-HGGs). Notably, 40% (4/10) of NBS-HGGs in children aged <3 years harboured an *NTRK* gene fusion [91]. Different fusions involving *NTRK* genes have also been reported in low-grade gliomas (Figure 2). Low-grade neuroepithelial tumours (LGNTs) are a diverse group of CNS tumours presenting in children and young adults; pilocytic astrocytomas are the most common LGNT seen in children. Jones et al. used whole-genome sequencing to analyse 96 pilocytic astrocytomas and identified two novel *NTRK2* gene fusions (*QKI-NTRK2* and *NACC2-NTRK2*) in three samples [92]. Qaddoumi et al. also utilised whole-genome sequencing to analyse 91 less common LGNTs and identified two tumours harbouring *NTRK2* translocations, including a novel *SLMAP-NTRK2* gene fusion found in a case of parietal ganglioglioma [93]. *NTRK* rearrangements have also been reported in diffuse leptomeningeal glioneuronal tumours [94]; rare CNS neoplasms that were included in the 2016 update of the World Health Organization classification [95]. In addition, cancers that can harbour *NTRK* gene fusions, such as lung cancers and melanomas, have a proclivity for CNS metastases [27, 96].

##### Spitzoid tumours/melanoma

Various translocations involving *NTRK1* or *NTRK3* have been reported in spitzoid melanocytic neoplasms as well as in compound Spitz nevi [97–99]. More recently, an NGS analysis was carried out by Lezcano et al. in order to assess the frequency of *NTRK* gene rearrangements in non-spitzoid metastatic melanomas. Among 751 cases, they identified three cutaneous primary melanomas (3/395; 0.8%) and one mucosal/paramucosal melanoma (1/113; 0.9%) harbouring *NTRK1* or *NTRK2* gene fusions [100].

##### Other tumour types

TRK fusions have also been reported in intrahepatic cholangiocarcinomas [101], breast cancer [102], quadruple wild-type (*ETV6*-*NTRK3*) gastrointestinal stromal tumours [103, 104], gallbladder adenocarcinomas [73], pancreatic carcinomas [105], sinus-nasal low-grade non-intestinal-type adenocarcinomas [106] and neuroendocrine tumours of the small bowel [107]. In addition to being present in solid tumours, *NTRK* gene fusions are also detected in acute lymphoblastic leukaemia (ALL) [108] and acute myeloid leukaemia [109] at a frequency of <5% [6].

### Preclinical and clinical evidence that *NTRK* gene fusions are oncogenic drivers

Preclinical studies with inhibitors of TRK proteins have further substantiated the role of *NTRK* gene fusions as oncogenic drivers. Mouse models of genetically engineered *NTRK* gene fusion-positive cancers have been shown to develop highly aggressive tumours. Two such studies involved a conditional knock-in model of carrying the *Etv6*-*NTRK3* gene fusion [109] and a chromosomal engineered glioma model harbouring the *Bcan-Ntrk1* gene fusion [110]. In both models, the tumours were effectively controlled using TRK inhibitors, indicating that the TRK fusion protein was implicated in the proliferation and survival of tumour cells. In a separate *in vitro* study, analysis of CRC cell lines revealed *NTRK1* overexpression that was associated with gene translocation. When this gene was suppressed through the use of short interfering RNA or TRKA inhibition, the ensuing reduction in protein expression or activity significantly impaired cell growth and increased apoptosis, suggesting functional dependency [111]. Furthermore, studies in mice demonstrated that conditional expression of an *Etv6*-*NTRK3* gene fusion was sufficient to initiate mammary tumourigenesis [112]. Importantly, *NTRK* gene fusions appear to be mutually exclusive to other gene alterations, suggesting that they may act as the sole oncogenic drivers in the tumours that harbour them [48, 82, 113].

Additional preclinical and clinical studies of tyrosine kinase inhibitors have provided further evidence of *NTRK* gene fusions as oncogenic drivers. Entrectinib (RDX-101, NMS-P626), a multikinase inhibitor, was shown to suppress TPM3-TRKA protein phosphorylation in mice with CRC harbouring a *TPM3-NTRK1* gene fusion [43], and further showed efficacy in three clinical trials including patients with *NTRK* gene fusions [114, 115]. Larotrectinib is a highly selective TRK inhibitor recently approved by the US Food and Drug Administration* for the treatment of adult and paediatric patients with solid tumours that harbour an *NTRK* gene fusion. Larotrectinib inhibited fusion protein signalling, *in vitro* proliferation and *in vivo* tumour growth in models derived from human cancer cells harbouring *NTRK* gene fusions [80, 97], as well as demonstrated clinical efficacy and safety in three clinical trials [46, 116, 117]. Resistance to larotrectinib and entrectinib can occur through the development of *NTRK* gene mutations, which involves amino acid substitutions in the solvent-front, gatekeeper residues of the *NTRK* genes (*NTRK1* p. G667C, *NTRK3* p. G696A) and xDFG motif substitutions [114, 118]. Second-generation TRK inhibitors, such as selitrectinib (BAY 2731954, LOXO-195), are under clinical development based on their ability to overcome acquired resistance mediated by these acquired recurrent mutations [114].

Other *NTRK* alterations, such as mutations, amplifications and mRNA overexpression, were found in ∼14% of 13 467 adult and paediatric pan-cancer tumour samples obtained from The Cancer Genome Atlas and the St Jude PeCan database [119]. *NTRK* mutations occur less frequently than amplifications or mRNA overexpression [119], but may be enriched in MSI-high CRCs [77]. These *NTRK* mutations are different from the acquired mutations described as a resistance mechanism to TRK inhibitors; as expected, the known acquired *NTRK* mutations that confer resistance were not observed in any of the 13 467 treatment-naïve tumours [119]. *NTRK* point mutations themselves are generally not activating oncogenic events [120] and have limited response to larotrectinib, as demonstrated in a phase I clinical trial of larotrectinib [117] where none of the patients with *NTRK* point mutations had an objective response to larotrectinib; in contrast, objective responses were seen in seven of eight patients with tumours harbouring *NTRK* gene fusions. The oncogenic role of TRK overexpression and *NTRK* gene amplification also remains unclear [6]. In the same trial with larotrectinib, one patient with a tumour harbouring an *NTRK1* gene amplification had a single 11 mm target lesion shrink by 5 mm (45.5%). The duration of response for this patient was 3.7 months, whereas in the patients with TRK fusion cancer the median duration of response had not been reached at a median follow-up of 26.9 months [117].

## Lessons learned

*NTRK* gene fusions can be drivers of cancer progression and, as such, their oncogenic products can be therapeutically targeted. Specific *NTRK* gene fusions have been identified in various tumours and can be found with high prevalence in certain rare adult and paediatric tumour types, even becoming a defining diagnostic feature, and at low prevalence in most common cancers. Advances in both *NTRK* gene fusion detection and targeted therapies to inhibit TRK are changing the diagnostic and therapeutic landscape of treatment of these cancers [46, 96].

## Footnotes

* Note added in proof: The European Medicines Agency granted marketing authorisation for larotrectinib on 23 September 2019 as monotherapy for the treatment of adult and paediatric patients with solid tumours that display a neurotrophic tyrosine receptor kinase (*NTRK*) gene fusion, and who have disease that is locally advanced, metastatic or where surgical resection is likely to result in severe morbidity, and who have no satisfactory treatment options.
